# COVID19-associated cardiomyocyte dysfunction, arrhythmias and the effect of Canakinumab

**DOI:** 10.1371/journal.pone.0255976

**Published:** 2021-08-19

**Authors:** Sanzio Dimai, Lukas Semmler, Ashok Prabhu, Harald Stachelscheid, Judith Huettemeister, Sandra C. Klaucke, Philipp Lacour, Florian Blaschke, Jan Kruse, Abdul Parwani, Leif-Hendrik Boldt, Lars Bullinger, Burkert M. Pieske, Frank R. Heinzel, Felix Hohendanner

**Affiliations:** 1 Department of Internal Medicine and Cardiology, Charité University Medicine, Berlin, Germany; 2 Institut für Physiologie und Pathophysiologie, Paracelsus Medizinische Privatuniversität, Nürnberg, Germany; 3 German Center for Cardiovascular Research (DZHK), Partner Site Berlin, Berlin, Germany; 4 Berlin Institute of Health (BIH) at Charité Universitätsmedizin Berlin, Stem Cell Core, Berlin, Germany; 5 Imperial College London, Hammersmith Hospital, London, England, United Kingdom; 6 Department of Nephrology and Medical Intensive Care, Charité-Universitätsmedizin Berlin, Berlin, Germany; 7 Department of Hematology, Oncology and Tumorimmunology, Charité University Medicine, Berlin, Germany; 8 Department of Internal Medicine and Cardiology, German Heart Center Berlin, Berlin, Germany; Scuola Superiore Sant’Anna, ITALY

## Abstract

**Background:**

Cardiac injury associated with cytokine release frequently occurs in SARS-CoV-2 mediated coronavirus disease (COVID19) and mortality is particularly high in these patients. The mechanistic role of the COVID19 associated cytokine-storm for the concomitant cardiac dysfunction and associated arrhythmias is unclear. Moreover, the role of anti-inflammatory therapy to mitigate cardiac dysfunction remains elusive.

**Aims and methods:**

We investigated the effects of COVID19-associated inflammatory response on cardiac cellular function as well as its cardiac arrhythmogenic potential in rat and induced pluripotent stem cell derived cardiomyocytes (iPS-CM). In addition, we evaluated the therapeutic potential of the IL-1β antagonist Canakinumab using state of the art in-vitro confocal and ratiometric high-throughput microscopy.

**Results:**

Isolated rat ventricular cardiomyocytes were exposed to control or COVID19 serum from intensive care unit (ICU) patients with severe ARDS and impaired cardiac function (LVEF 41±5%; 1/3 of patients on veno-venous extracorporeal membrane oxygenation; CK 154±43 U/l). Rat cardiomyocytes showed an early increase of myofilament sensitivity, a decrease of Ca^2+^ transient amplitudes and altered baseline [Ca^2+^] upon exposure to patient serum. In addition, we used iPS-CM to explore the long-term effect of patient serum on cardiac electrical and mechanical function. In iPS-CM, spontaneous Ca^2+^ release events were more likely to occur upon incubation with COVID19 serum and nuclear as well as cytosolic Ca^2+^ release were altered. Co-incubation with Canakinumab had no effect on pro-arrhythmogenic Ca^2+^ release or Ca^2+^ signaling during excitation-contraction coupling, nor significantly influenced cellular automaticity.

**Conclusion:**

Serum derived from COVID19 patients exerts acute cardio-depressant and chronic pro-arrhythmogenic effects in rat and iPS-derived cardiomyocytes. Canakinumab had no beneficial effect on cellular Ca^2+^ signaling during excitation-contraction coupling. The presented method utilizing iPS-CM and in-vitro Ca^2+^ imaging might serve as a novel tool for precision medicine. It allows to investigate cytokine related cardiac dysfunction and pharmacological approaches useful therein.

## Introduction

The current Sars-CoV-2 pandemic affects health care systems worldwide in an unprecedented way, resulting in high morbidity and mortality. Cardiac injury occurs in almost 20% of patients during hospitalization and mortality is particularly high in these patients [1]. Troponin elevations are a frequently reported finding [2] and arrythmias are linked to ICU admission in up to 12% of patients, while acute respiratory distress syndrome (ARDS) and sepsis occur in up to 29% of hospital admissions [3]. In addition, a considerable number of patients present with cardiovascular comorbidities (up to 15%) worsening their overall outcome. Systemic inflammation and cytokine release are a hallmark feature of the disease [4].

However, the role of COVID19 associated cytokine-storm for concomitant cardiac dysfunction [5] remains elusive. In addition, potential beneficial effects of immunomodulation to decrease circulatory cytokines with their assumed negative impact on cardiac contractile function have not yet been established. Cardiac mechanical and electrical function are disturbed during heart failure with reduced ejection fraction (HFrEF) leading to acute decompensation, ventricular tachyarrhythmias and sudden cardiac death. HFrEF is a feared complicator of septic conditions on the ICU and associated with increased mortality [6]. Direct and indirect triggers for HFrEF include infectious viral diseases. It is widely known that a variety of viruses causes inflammatory cardiomyopathy leading to impaired function [7].

However, besides direct infection of myocytes with cardiotropic viruses, septic cardiomyopathy is a common denominator of severely affected ICU patients that suffer from a variety of viral infections. Septic cardiomyopathy has been associated with an altered interleukin profile in peripheral blood samples [8] and interleukins like TNFα and IL-1β have been shown to depress myocardial contractile function [9]. Clinical reports indicate that critically ill COVID19 patients develop sepsis and ARDS at days 10 and 12, which is paralleled by a surge of cytokines (i.e. IL-1, IL-2, IL-6, IL-7, IL-10, GSCF, IP-10, MCP1, MIP1A, TNFα) known to be involved in septic cardiomyopathy and clinical deterioration [3]. Several groups have suggested early-on to utilize immunosuppressant therapy for the treatment of COVID19 associated sepsis. While the IL-6 receptor blocker Tocilizumab administered to COVID19 patients in cytokine release syndrome has not shown any benefit in terms of survival in a very recent multicenter randomized controlled trial [10–12], the IL-1 receptor blockers Anakinra and Canakinumab conferred a significant survival benefit in patients with septic hyperinflammation [10, 13, 14].

The specific effects of anti-inflammatory treatment on the concomitant cardiac contractile and electric function [5] in the context of COVID19 remain elusive. We investigated the effect of the COVID19 associated cytokine-release on cardiac function in-vitro using a rat model and induced pluripotent stem-cell derived cardiomyocytes. For that purpose, we established an in-vitro setup to study potential effects of anti-inflammatory treatment on cardiac dysfunction as exemplified by the IL-1β inhibitor Canakinumab.

## Material and methods

### Chemicals and solutions

Chemicals were obtained from Sigma Aldrich (St. Louis, MO, USA) if not noted otherwise. Fluorescent dyes Fluo-4 and Fura-2 AM were obtained from Thermo Fisher Scientific (Waltham, MA, USA). Normal Tyrode (NT) solution consisted of (in mM): 30 NaCL, 4 KCl, 2 CaCl, 1 MgCl2, 10 Glucose, 10 HEPES and was pH adjusted to 7.4 with NaOH.

### Patient serum

The study was conducted at the Charité Campus Virchow Hospital between 04/2020 and 12/2020 in accordance with the local ethics committee approval (EA2/066/20; pa-COVD19/Charité; “Ethikausschuss am Campus Virchow Klinikum”; Head: PD Dr. E. Kaschina). Patients provided informed written consent to have data from their medical records used in research and to participate in this study. Patient serum was acquired from either healthy control donors (n = 3) or SARS-CoV-2 positive patients (n = 12) with severe acute respiratory distress syndrome (i.e. Horowitz index <300; see Table 1 for patient characterization) immediately upon intensive care unit (ICU) admission. Serum was obtained using a serum separator tube upon immediate cooled centrifugation and stored at -80°C. Patient characteristics are shown in Table 1 and ELISAs were performed for interleukin(IL)-1β, IL-6 and TNFα as per kit instructions (Invitrogen/Thermo Fisher Scientific, USA). C-reactive protein (CRP), procalcitonin (PCT), creatinine kinase (CK) and hemoglobin (Hb) concentrations were obtained using the clinical routine laboratory infrastructure at Charité University Medicine Berlin.

**Table 1:** Patient characteristics of the study cohort. [15, 16].

	COVID19 (n = 12)	Control (n = 3)	Reference Range
Sex (%)	♂: 75 / ♀: 25	♂: 100/ ♀: 0	-
Age (ys)	64±4	35±6	-
LVEF (%)	41±5	58±2	55–70
VV-ECMO (%)	33	0	-
CVVHD (%)	58	0	-
Anticoagulation (%)	100	0	-
Glucorticoids (%)	67	-	-
Antibiotics (%)	92	-	-
Hb (mg/dl)	9.1±0.3	-	♂: >13.5 / ♀: >12
CK (U/l)	154±43	-	♂: <170 / ♀: <145
CRP (mg/dl)	173±30	-	<5
PCT (μg/l)	6.6±2.4	-	<0.5
Catecholamines (μg/kg/h)	0.06625±0.03	0	-
Horowitz-Ratio	220±24	-	>500
IL-6 (pg/ml)	12±7	0.5±0.1	<4.5 ^(Todd et al; 15)^
IL-1β (pg/ml)	2±1	< 0.1	0.3–1.4 ^(Iorio et al; 16)^
TNFα (pg/ml)	23±1	18±8	<2.5 ^(Todd et al; 15)^

### Animal in-vitro experiments

All animal experiments were approved by local authorities and performed in accordance with local guidelines (local ethical committee approval T0060-15, LAGeSo Berlin). Ventricular cardiomyocytes were isolated from 12 to 16 weeks old, male Sprague Dawley rats as previously described [17]. In short, rats were sacrificed using cervical dislocation, hearts were excised, the aorta was cannulated and mounted on a Langendorff apparatus. Perfusion with a calcium (Ca^2+^) free solution for 2 min was followed by enzymatic digestion in a solution containing 20 μM Ca^2+^ and 75μg/ml Liberase (Sigma-Aldrich (St. Louis, MO, USA) for 12 to 15 min. The left ventricle was removed from the heart, the tissue was dissected, filtered and washed and Ca^2+^ was introduced stepwise. Isolated cardiomyocytes were kept in NT solution between experiments.

Isolated adult rat ventricular cardiomyocytes were used for two different sets of experiments: First, we investigated effects upon ultra-short term (i.e. 15 min) and second upon short-term (i.e. 60 min) incubation in either patient or control serum. For the ultra-short term study, aiming at acute receptor mediated effects, a subset of ventricular cardiomyocytes was loaded with Fura-2 AM at 1μM for 15 min and plated on laminin-coated coverslips in NT solution. The coverslips were mounted on a computer-controlled-microscope (CytoCypher MultiCell System, CytoCypher BV, Netherlands), cells were electrically stimulated (1 and 3 Hz field-stimulation; Myopacer, Ionoptix) and fluorescence (excitation at 340 nm and 385 nm, emission collected at 510±40) and sarcomere shortening were recorded at 37°C during steady state. Subsequently, NT solution was replaced by solution containing either patient or control serum diluted to 10% and cells were incubated for 15 min. Fluorescence signal and contractility were measured in a paired fashion (cells individual position was stored digitally) while cells remained in 10% serum solution.

For the short term study and to mimic in-vivo conditions as closely as possible, ventricular cardiomyocytes were incubated for 60 min in either patient or control serum diluted to 50% at room temperature. Cells were then removed from the serum solution, washed twice, plated on laminin-coated coverslips and loaded with Fura-2 AM at 1 μM for 15 min. The additional wash-step was introduced to mitigate interferences by the serums autofluorescence and heterogeneity in local fluorescence.

Fluorescence and contractility were assessed as described above. Recorded fluorescence and contractility data were analyzed using the software Cytosolver Desktop (CytoCypher MultiCell System, CytoCypher BV, Netherlands).

### Human induced pluripotent stem cells experiments

iPS-derived cardiomyocytes (iPS-CM) were generated from the previously established hiPSC line BIHi004-A generated from cells of a healthy donor (see Hossini et al. [18] and https://hpscreg.eu/cell-line/BIHi004-A for further details) using an in-house optimized protocol established at the BIH Stem Cell Core that is based on the protocol described by Lian et al [19]. IPS-CM were incubated for 24 hours in either patient or control (healthy donors) serum diluted to 10% with maintenance medium (RPMI 1640 Thermo Fisher Scientific, USA with B27 Supplement (50X) Thermo Fisher Scientific, USA) at 37.5°C. Incubation was performed with or without 10μg/ml of the monoclonal anti-IL-1β antibody Canakinumab (Novus Biologicals, Colorado, USA) present. Cells were subsequently loaded with Fluo-4-AM and fluorescence was measured using confocal line-scan imaging (Zeiss LSM 800, excitation at 488 nm, emission collected at > 515 nm). Clusters of cells were identified and the scan line was placed along the maximal diameter of at least one cell with a pixel-size of 0.12 μm. Cells were paced at 1 Hz (field-stimulation) and images were acquired either during steady-state or immediately upon cessation of electrical stimulation as indicated. Local Ca^2+^ transients were calculated as F / F_0_ from regions that were identified to be cytosolic or nuclear using 2D confocal images of cell-clusters. Spontaneous Ca^2+^ release measurements were obtained during a 10 sec period upon termination of electrical stimulation.

### Statistical analysis

Experiments with serum were performed in a non-pooled fashion for each individual patient. All data is presented as mean ± standard error mean and analysis was performed in a blinded-fashion. Wherever feasible, individual data points are displayed, otherwise sample sizes are provided. Parts of the data analysis were conducted in R (version 3.6.1, R Core Team (2018)), scripts are fully available upon request. GraphPad Prism was used for statistical inference and plotting (GraphPad Software, San Diego, California USA). To test for group differences, for data with two groups student’s t-test or Kruskal-Wallis One Way ANOVA on Ranks in non-normal distributed data was used. For data with more than two groups ANOVA analysis was performed. A p<0.05 indicates significant statistical difference.

## Results

Patients included in the study were mainly male (n = 12; 75% male), at an age of 64 ± 4 years and showed an impaired left ventricular ejection fraction of 41 ± 5%. In this severely diseased patient cohort, 33% of patients were treated with veno-venous ECMO therapy and markers for cardiac injury and inflammation were increased (mean CK 154±43 U/l, mean CRP 173±30 mg/dl; Table 1).

To test the effects of patient serum on cardiac excitation-contraction coupling mimicking different clinical scenarios of severe COVID19 mediated ARDS, cardiomyocytes were incubated for an ultra-short, short or long term period in either patient or control serum.

**Fig 1:**
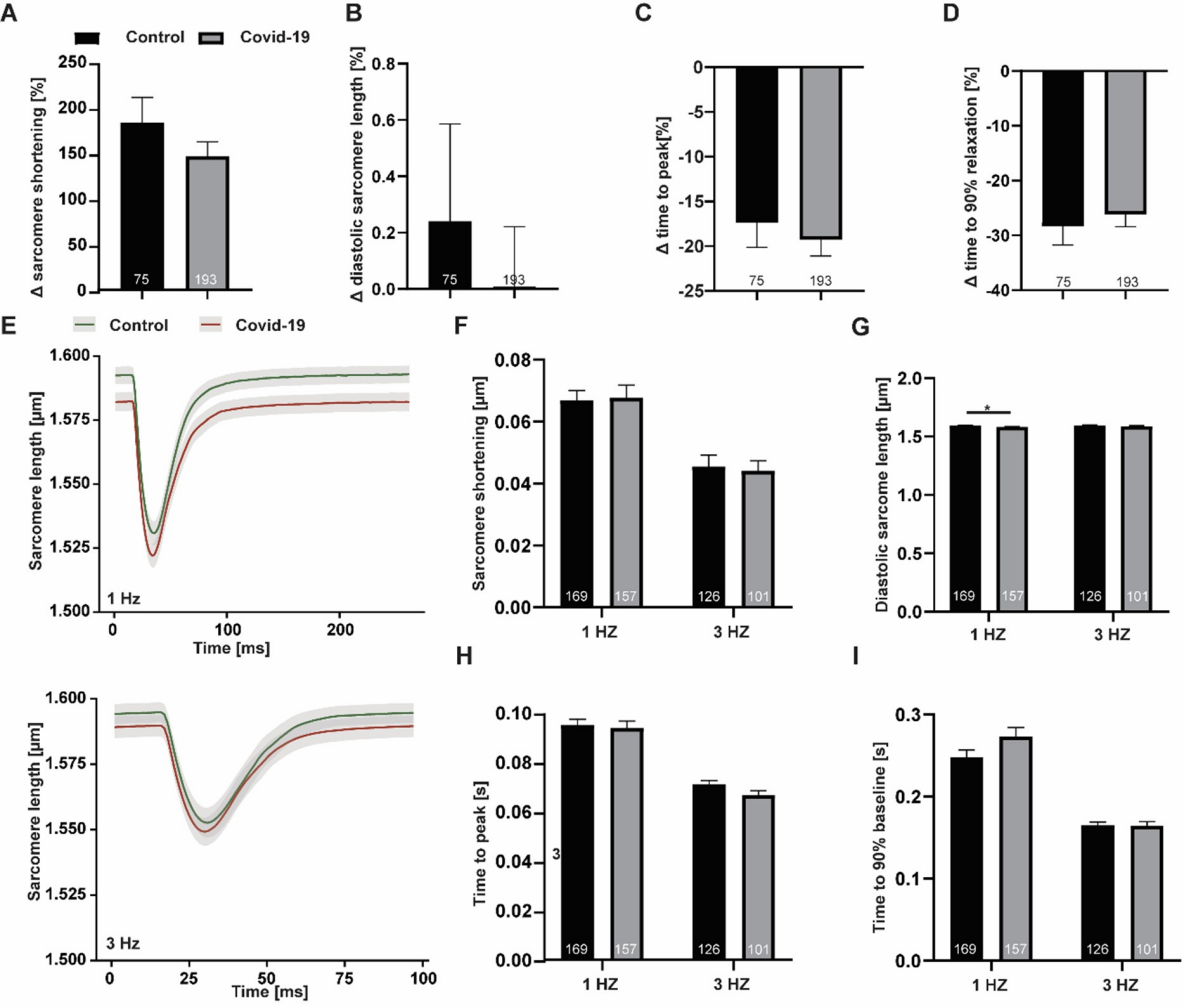
Excitation-contraction coupling in rat cardiomyocytes after 15 (A-D) and 60 (E-I) minutes incubation in either control (black) or COVID19 (grey) plasma, respectively. (A-D) Depicted as change after incubation in percent of initial value. (A) relative sarcomere shortening amplitude. (B) diastolic sarcomere length, (C) time to peak of sarcomere shortening and (D) time to 90% of relaxation. (E) Sarcomere shortening averaged over all cells per group at 1 Hz (upper) and 3 Hz (lower) electric stimulation and 1 mM extracellular [Ca2+]. Related data of (F) sarcomere shortening amplitude, (G) diastolic sarcomere length, (H) time to sarcomere shortening peak and (I) time to 90% sarcomere relaxation. *p<0.05.

First we performed experiments using ratiometric imaging to determine ultra-short term (15 minutes incubation) and short term (60 minutes incubation) effects on rat ventricular cardiomyocyte contractile function during excitation-contraction coupling. When exposing isolated adult rat ventricular cardiomyocytes to COVID19 serum the amplitude and kinetics of sarcomere shortening were not affected at 15 minutes. Interestingly, in these paired measurements, we observed an augmentation of contractile function upon addition of control and patient serum alike (difference of amplitude after and before incubation: +183% ± 27% vs +145% ± 16% (control vs patient), n = 75 vs 193, p = 0.23). After 60 minutes at either 1 Hz or 3 Hz maximum sarcomere shortening was still unaffected (1Hz: 0.06. ± 0.003 vs 0.06 ± 0.004 (control vs patient), μm, n = 167 vs 157, p = 0.87; 3 Hz: 0.045 ± 0.003 vs 0.044 ± 0.003 (control vs patient), μm, n = 126 vs 101, p = 0.78). Although we did see a slight shortening of diastolic sarcomere length with patient serum at 1 Hz, this was not seen at 3 Hz (1 Hz: 1.59 ± 0.003 vs 1.58 ± 0.003 (control vs patient), μm, n = 169 vs 157, p = 0.03; 3 Hz: 1.59 ± 0.003 vs 1.58 ± 0.004 (control vs patient), μm, n = 126 vs 101, p = 0.36). In addition, contraction and relaxation kinetics were unaltered (Fig 1).

In a next step, we determined Ca^2+^ transient amplitudes upon short term exposure to COVID19 serum.

**Fig 2:**
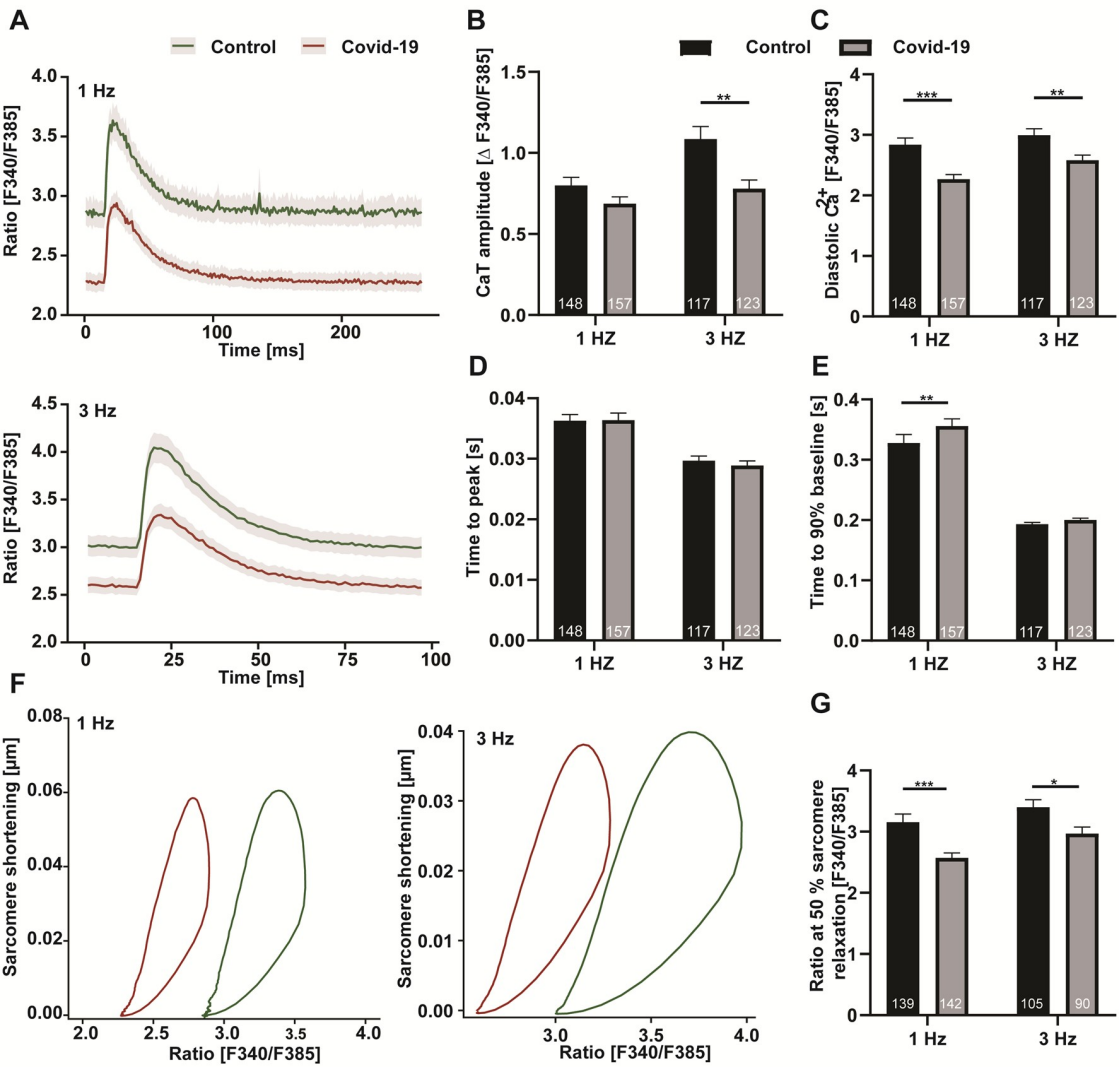
Ca^2+^ signaling during excitation-contraction coupling (rat) after 60 minutes incubation with control (black) or COVID19 (grey) serum. (A) Ca^2+^ transients averaged over all cells per group at 1 Hz (upper) and 3 Hz (lower) electric stimulation and 1 mM extracellular [Ca2+]. Related data of (B) Ca^2+^ transient amplitude, (C) diastolic Ca^2+^, (D) time to Ca^2+^ transient peak and (E) time to 90% relaxation. (F) Cardiomyocyte Ca^2+^-sarcomere loop; all-cell/group average at 1 Hz (left) and 3 Hz (right). (G) [Ca^2+^] at 50% sarcomere relaxation. *p<0.05, ** p < 0.005, *** p < 0.001.

Incubation with patient serum significantly reduced Ca^2+^ transient amplitudes at 1 Hz in comparison to control serum (0.79 ± 0.04 vs 0.68 ± 0.04 (control vs patient), arbitrary units, n = 148 vs 157, p = 0.08) with an even more pronounced effect at 3 Hz (1.08 ± 0.07 vs 0.77 ± 0.05. (control vs patient), arbitrary units, n = 117 vs 123, p = 0.01). The positive inotropic cellular response to the increase in stimulation frequency was almost completely blunted with patient serum (difference of amplitude 3 Hz to 1 Hz: +0.29 vs +0.09 (control vs patient)). The decrease in amplitude was accompanied by a reduction of diastolic Ca^2+^ at both frequencies (1Hz: 2.83 ± 0.11 vs 2.26 ± 0.07 (control vs patient), arbitrary units, n = 148 vs 157, p< 0.001; 3 Hz: 2.99 ± 0.1 vs 2.58 ± 0.08 (control vs patient), arbitrary units, n = 117 vs 123, p = 0.002) (Fig 2).

To further investigate the impact of COVID19 serum on short-term cardiomyocyte function, we computed Ca^2+^-contractility loops and calculated the Ca^2+^ concentration at 50% sarcomere relaxation, a phase of the loop where the Ca^2+^-contractility relation is almost linear (Fig 2). This parameter has previously been described as a measure of microfilament Ca^2+^ sensitivity [20]. With patient serum, it decreased significantly, indicating an increased Ca^2+^ sensitivity (1 Hz: 3.15 ± 0.13 vs 2.57 ± 0.07 (control vs patient), arbitrary units, n = 139 vs 142, p <0.001; 3 Hz: 3.58 ± 0.16 vs 2.97 ± 0.10 (control vs patient), arbitrary units, n = 105 vs 90, p = 0.01) [21].

**Fig 3:**
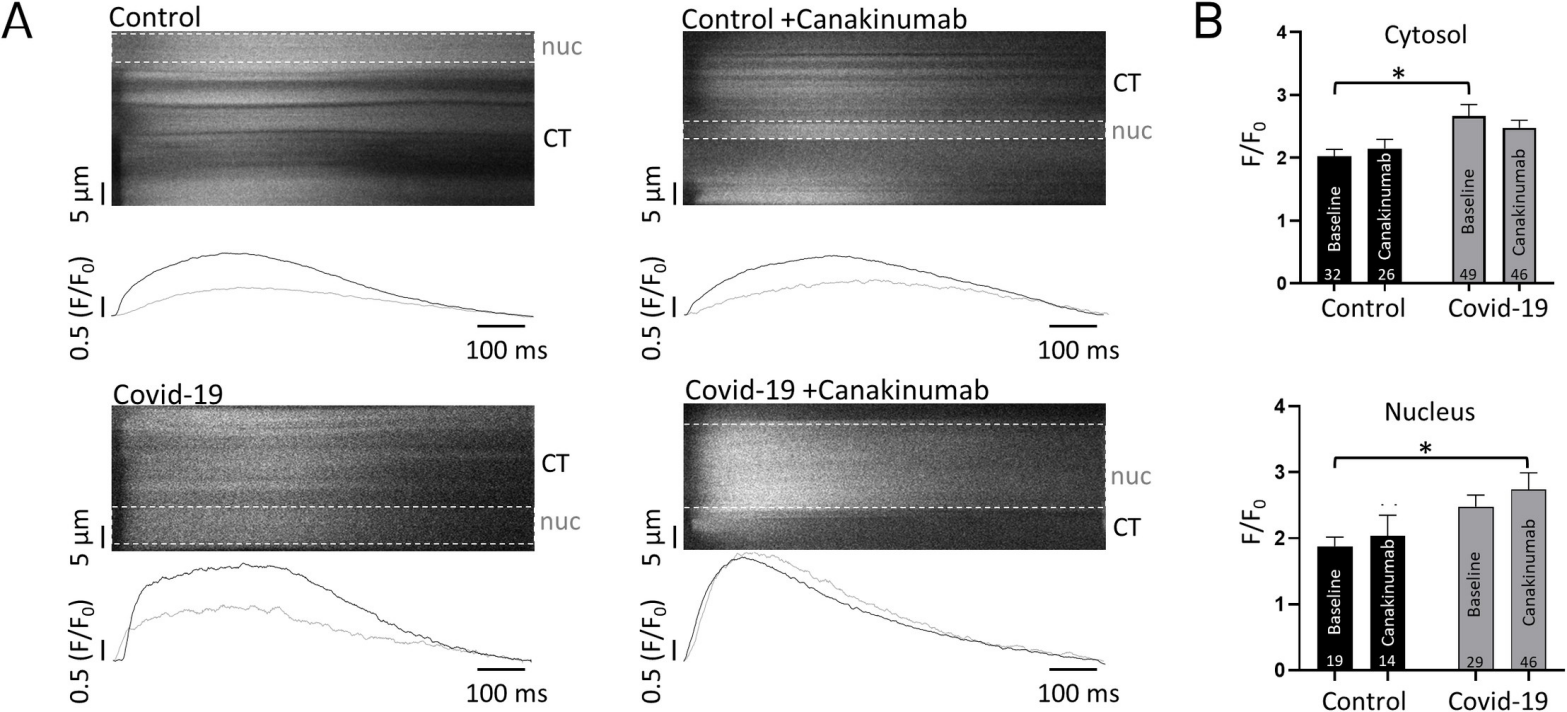
Ca^2+^ transients during electrical stimulation of iPS-CM upon addition of COVID19 serum. (A) Example Ca^2+^ transients as obtained after 24 hours incubation with serum +/- Canakinumab; iPS-CM during 1Hz electrical field stimulation. (B) Quantification of cytosolic (top) and nuclear (bottom) peak Ca^2+^ transient amplitudes. *p<0.05.

To overcome issues with longevity of isolated adult cardiomyocytes and to test our pharmacological approach with IL-1β inhibition in a setting that resembles human cardiomyocytes more closely, we used iPS-CM for our long term experiments. As shown in Fig 3, after 24 hours incubation Ca^2+^ signaling during excitation-contraction signaling was significantly augmented in the cytosolic compartment, indicating profound changes of the Ca^2+^ release machinery. However, addition of Canakinumab had no significant effect on Ca^2+^ transient amplitudes (Fig 3).

**Fig 4:**
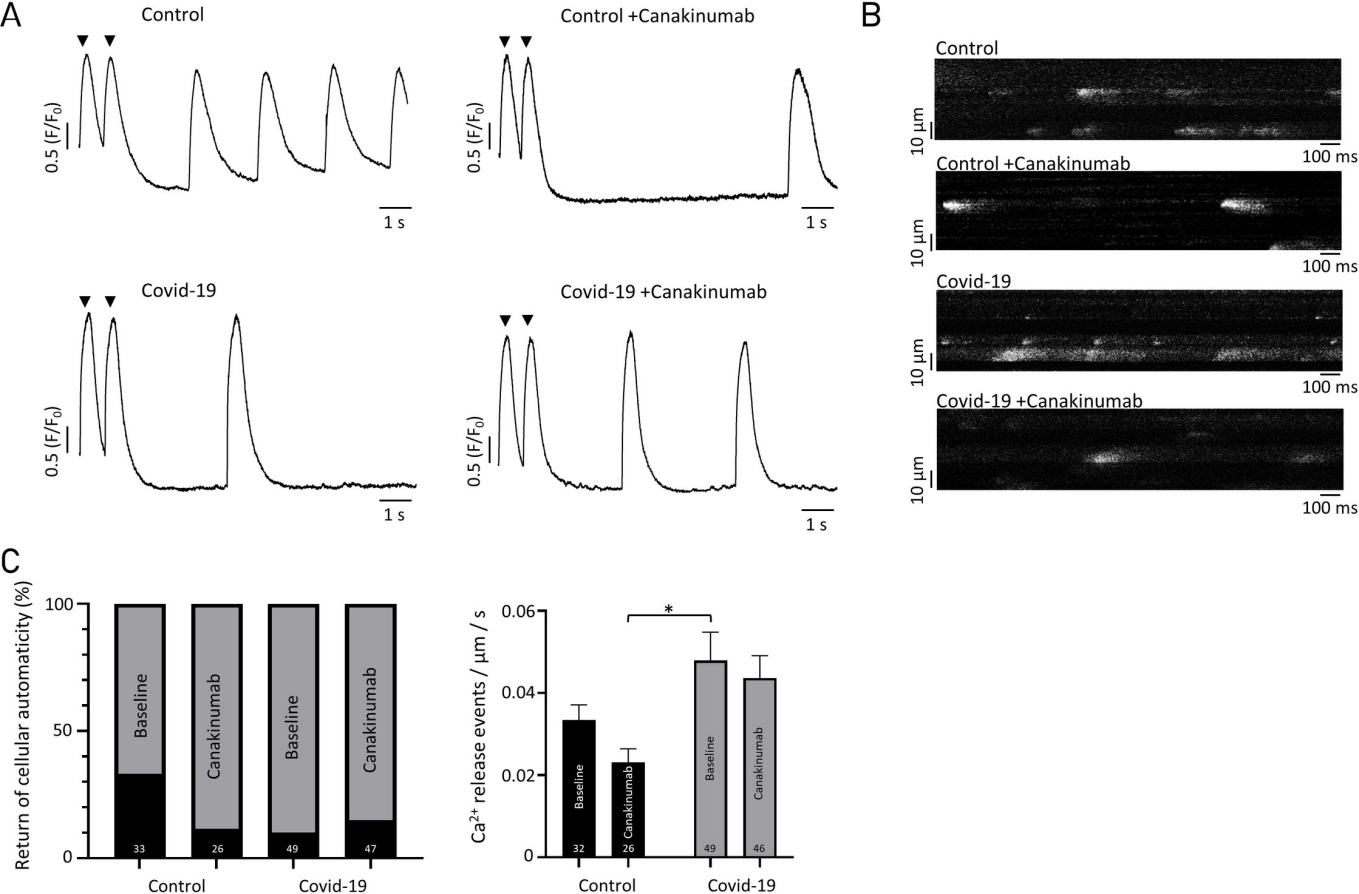
Arrhythmogenic Ca^2+^ release in iPS-CM upon addition of COVID19 serum and treatment with Canakinumab. (A) Example for stimulated and spontaneous (“cellular automaticity”) Ca^2+^ transients in iPS-CM as obtained after 24 hours incubation with serum +/- Canakinumab; Electrical field-stimulation with 1 Hz as indicated (black arrows). (B) Spontaneous Ca^2+^ release events in iPS-CM upon prolonged 1 Hz electrical stimulation. (C) Quantification of cellular automaticity and Ca^2+^ release events in iPS-CM. *p<0.05.

Long-term incubation with COVID19 patient serum also had effects on cellular arrhythmogenic potential: Ca^2+^ waves showed a trend to occur more often upon incubation with patient serum as compared to control (0.22 ± 0.04 vs 0.37 ± 0.04 (control vs patient), waves/sec, n = 32 vs 49, p = 0.079) and spontaneous Ca^2+^ release events (i.e. Ca^2+^ sparks) were increased upon addition of COVID serum (Fig 4). Spontaneous action potential activity of iPS-CM is dependent on the proper function of membrane ion channels and cell-cell interactions [22]. We measured the return of spontaneous action potential generation upon prolonged (i.e. >5 min) electrical field-stimulation. Cells incubated with control serum had a significantly more frequent return to cellular automaticity after the end of stimulation than those incubated with patient serum (long term incubation). However, Canakinumab did not affect arrhythmogenic potential nor the return of cellular automaticity (Fig 4C).

## Discussion

The present study used a non-selective approach to study the effect of COVID19 associated cytokine release on cardiac in-vitro function in ICU patients. Indeed, serum derived from COVID19 patients was associated with acute cardio-depressant and chronic pro-arrhythmogenic effects in both, rat and iPS-CM. This impairment of Ca^2+^ release might be associated with the serum protein and cytokine profile of our set of ICU COVID19 patients: It has been shown that levels of IL-1, TNFα, IL6 and other cytokines are significantly increased in patients resembling our clinical collective with COVID19 [23–25]. However, of note, 92% of the patients included in this study received antibiotic treatment at the time of sample acquisition. This indicates bacterial superinfection and might be a confounding factor in this collective. 67% of the included patients also received glucocorticoids potentially altering the cytokine profile. IL-1 and TNFα are known pro-inflammatory cytokines with negative effects on cellular Ca^2+^ signaling during excitation contraction coupling in septic cardiomyopathy [26]. Even though poorly correlated to survival in COVID19 [27], especially IL-1β has been shown to decrease the beta-adrenergic responsiveness of L-type Ca^2+^ channels in a cAMP-independent mechanism, potentially decreasing cellular inotropic response [28]. This is in line with our observed altered cytosolic [Ca^2+^] at 60 minutes serum incubation and further supported by results from Mitrokhin et al [29], associating electrical abnormalities and action potential prolongations with IL-1.

We detected a significant difference in Ca^2+^ sensitivity upon cellular exposure to COVID19 serum. Notably, among critically ill patients with COVID19, ventricular malign arrhythmias were found in 2.5% of patients and especially prevalent in non-survivors (RR: 3.8) [30]. As demonstrated by Huke et al., regional slowing of impulse propagation, which can subsequently trigger reentrant activation and arrhythmias, is associated with altered Ca^2+^ sensitivity [31]. Our observed changes in Ca^2+^ sensitivity might contribute to the arrhythmogenic potential in severe COVID19 as this relation has been previously described for other models of septic cardiomyopathy [32].

Arrhythmic Ca^2+^ release was observed especially after long-term exposure to COVID19 serum. In the present study, the IL-1β blocker Canakinumab was used to potentially mitigate these in-vitro effects, as Canakinumab has been shown to improve outcomes in mild or severe COVID19 pneumonia in early clinical trials with limited numbers of patients [33]. However, chronic co-incubation with Canakinumab had no beneficial effect on cellular Ca^2+^ signaling during excitation-contraction coupling or on electrical dysfunction in our iPS-CM model. This missing effect might be linked to our heterogeneous patient collective and sample acquisition in a real-life scenario–yet it is supported by a very recent randomized clinical trial with over 450 patients, indicating only little effect of Canakinumab on overall patient outcome (CAN-COVID; trial identifier: NCT04362813). Further clinical trials like the currently conducted three C study [34] are going to assess potential benefits of Canakinumab treatment in COVID19 patients limited to cardiac effects. The presented method utilizing iPS-CM and Ca^2+^ imaging allows to study acute and chronic cardiac effects in conditions associated with cytokine release. As exemplified in the current study using Canakinumab, this approach might also serve as an in-vitro “assay” to assess the effectiveness of different drugs in this particular setting.

In conclusion, serum derived from COVID19 patients exerts acute cardio-depressant and chronic pro-arrhythmogenic effects in rat and iPS-derived cardiomyocytes, respectively. Chronic co-incubation with Canakinumab had no beneficial effect on cellular Ca^2+^ signaling during excitation-contraction coupling. The presented method utilizing iPS-CM to screen for the effectiveness of different pharmacological approaches in the context of conditions associated with cytokine release might serve as a novel method for precision medicine.

## References

[1] Shi S, Qin M, Shen B, Cai Y, Liu T, Yang F, et al. Association of Cardiac Injury With Mortality in Hospitalized Patients With COVID-19 in Wuhan, China. JAMA Cardiol. 2020. doi: 10.1001/jamacardio.2020.0950. 32211816 PMC7097841

[2] Ruan Q, Yang K, Wang W, Jiang L, Song J. Clinical predictors of mortality due to COVID-19 based on an analysis of data of 150 patients from Wuhan, China. Intensive Care Med. 2020. doi: 10.1007/s00134-020-05991-x; PubMed Central PMCID: PMC7080116. 32125452 PMC7080116

[3] Zhou F, Yu T, Du R, Fan G, Liu Y, Liu Z, et al. Clinical course and risk factors for mortality of adult inpatients with COVID-19 in Wuhan, China: a retrospective cohort study. Lancet. 2020. doi: 10.1016/S0140-6736(20)30566-3. 32171076 PMC7270627

[4] Guan WJ, Ni ZY, Hu Y, Liang WH, Ou CQ, He JX, et al. Clinical Characteristics of Coronavirus Disease 2019 in China. N Engl J Med. 2020. doi: 10.1056/NEJMoa2002032. 32109013 PMC7092819

[5] Bonow RO, Fonarow GC, O’Gara PT, Yancy CW. Association of Coronavirus Disease 2019 (COVID-19) With Myocardial Injury and Mortality. JAMA Cardiol. 2020. doi: 10.1001/jamacardio.2020.1105. 32219362

[6] Jeong HS, Lee TH, Bang CH, Kim JH, Hong SJ. Risk factors and outcomes of sepsis-induced myocardial dysfunction and stress-induced cardiomyopathy in sepsis or septic shock: A comparative retrospective study. Medicine (Baltimore). 2018;97(13):e0263. doi: 10.1097/MD.0000000000010263; PubMed Central PMCID: PMC5895365. 29595686 PMC5895365

[7] Tschope C, Cooper LT, Torre-Amione G, Van Linthout S. Management of Myocarditis-Related Cardiomyopathy in Adults. Circ Res. 2019;124(11):1568–83. doi: 10.1161/CIRCRESAHA.118.313578 . 31120823

[8] Cirulis MM, Beesley SJ, Wilson EL, Stubben C, Olsen TD, Hirshberg EL, et al. The peripheral blood transcriptome in septic cardiomyopathy: an observational, pilot study. Intensive Care Med Exp. 2019;7(1):57. doi: 10.1186/s40635-019-0271-0; PubMed Central PMCID: PMC6813402. 31650252 PMC6813402

[9] Cain BS, Meldrum DR, Dinarello CA, Meng X, Joo KS, Banerjee A, et al. Tumor necrosis factor-alpha and interleukin-1beta synergistically depress human myocardial function. Crit Care Med. 1999;27(7):1309–18. doi: 10.1097/00003246-199907000-00018 . 10446825

[10] Mehta P, McAuley DF, Brown M, Sanchez E, Tattersall RS, Manson JJ, et al. COVID-19: consider cytokine storm syndromes and immunosuppression. Lancet. 2020. doi: 10.1016/S0140-6736(20)30628-0. 32192578 PMC7270045

[11] Fontes JA, Rose NR, Cihakova D. The varying faces of IL-6: From cardiac protection to cardiac failure. Cytokine. 2015;74(1):62–8. doi: 10.1016/j.cyto.2014.12.024 ; PubMed Central PMCID: PMC4677779. 25649043 PMC4677779

[12] Rosas IO, Brau N, Waters M, Go RC, Hunter BD, Bhagani S, et al. Tocilizumab in Hospitalized Patients with Severe Covid-19 Pneumonia. N Engl J Med. 2021. Epub 2021/02/26. doi: 10.1056/NEJMoa2028700; PubMed Central PMCID: PMC7953459. 33631066 PMC7953459

[13] Shakoory B, Carcillo JA, Chatham WW, Amdur RL, Zhao H, Dinarello CA, et al. Interleukin-1 Receptor Blockade Is Associated With Reduced Mortality in Sepsis Patients With Features of Macrophage Activation Syndrome: Reanalysis of a Prior Phase III Trial. Crit Care Med. 2016;44(2):275–81. doi: 10.1097/CCM.0000000000001402 ; PubMed Central PMCID: PMC5378312. 26584195 PMC5378312

[14] Abbate A, Toldo S, Marchetti C, Kron J, Van Tassell BW, Dinarello CA. Interleukin-1 and the Inflammasome as Therapeutic Targets in Cardiovascular Disease. Circ Res. 2020;126(9):1260–80. Epub 2020/04/24. doi: 10.1161/CIRCRESAHA.120.315937 . 32324502 PMC8760628

[15] Todd J, Simpson P, Estis J, Torres V, Wub AH. Reference range and short- and long-term biological variation of interleukin (IL)-6, IL-17A and tissue necrosis factor-alpha using high sensitivity assays. Cytokine. 2013;64(3):660–5. Epub 2013/10/17. doi: 10.1016/j.cyto.2013.09.018 . 24128872

[16] Di Iorio A, Ferrucci L, Sparvieri E, Cherubini A, Volpato S, Corsi A, et al. Serum IL-1beta levels in health and disease: a population-based study. ’The InCHIANTI study’. Cytokine. 2003;22(6):198–205. Epub 2003/08/02. doi: 10.1016/s1043-4666(03)00152-2 . 12890453

[17] Hohendanner F, Bode D, Primessnig U, Guthof T, Doerr R, Jeuthe S, et al. Cellular mechanisms of metabolic syndrome-related atrial decompensation in a rat model of HFpEF. J Mol Cell Cardiol. 2018;115:10–9. Epub 2018/01/01. doi: 10.1016/j.yjmcc.2017.12.012 . 29289652

[18] Hossini AM, Quast AS, Plotz M, Grauel K, Exner T, Kuchler J, et al. PI3K/AKT Signaling Pathway Is Essential for Survival of Induced Pluripotent Stem Cells. PLoS One. 2016;11(5):e0154770. Epub 2016/05/04. doi: 10.1371/journal.pone.0154770; PubMed Central PMCID: PMC4854383. 27138223 PMC4854383

[19] Lian X, Hsiao C, Wilson G, Zhu K, Hazeltine LB, Azarin SM, et al. Robust cardiomyocyte differentiation from human pluripotent stem cells via temporal modulation of canonical Wnt signaling. Proc Natl Acad Sci U S A. 2012;109(27):E1848–57. doi: 10.1073/pnas.1200250109 ; PubMed Central PMCID: PMC3390875. 22645348 PMC3390875

[20] Curl CL, Danes VR, Bell JR, Raaijmakers AJA, Ip WTK, Chandramouli C, et al. Cardiomyocyte Functional Etiology in Heart Failure With Preserved Ejection Fraction Is Distinctive-A New Preclinical Model. J Am Heart Assoc. 2018;7(11). doi: 10.1161/JAHA.117.007451; PubMed Central PMCID: PMC6015350. 29858360 PMC6015350

[21] Hobai IA, Edgecomb J, LaBarge K, Colucci WS. Dysregulation of intracellular calcium transporters in animal models of sepsis-induced cardiomyopathy. Shock. 2015;43(1):3–15. Epub 2014/09/05. doi: 10.1097/SHK.0000000000000261 ; PubMed Central PMCID: PMC4269564. 25186837 PMC4269564

[22] Kim JJ, Yang L, Lin B, Zhu X, Sun B, Kaplan AD, et al. Mechanism of automaticity in cardiomyocytes derived from human induced pluripotent stem cells. J Mol Cell Cardiol. 2015;81:81–93. Epub 2015/02/04. doi: 10.1016/j.yjmcc.2015.01.013 ; PubMed Central PMCID: PMC4409767. 25644533 PMC4409767

[23] Rowaiye AB, Okpalefe OA, Onuh Adejoke O, Ogidigo JO, Hannah Oladipo O, Ogu AC, et al. Attenuating the Effects of Novel COVID-19 (SARS-CoV-2) Infection-Induced Cytokine Storm and the Implications. J Inflamm Res. 2021;14:1487–510. Epub 2021/04/24. doi: 10.2147/JIR.S301784 ; PubMed Central PMCID: PMC8057798. 33889008 PMC8057798

[24] van de Veerdonk FL, Netea MG. Blocking IL-1 to prevent respiratory failure in COVID-19. Crit Care. 2020;24(1):445. Epub 2020/07/20. doi: 10.1186/s13054-020-03166-0; PubMed Central PMCID: PMC7411343. 32682440 PMC7411343

[25] Costela-Ruiz VJ, Illescas-Montes R, Puerta-Puerta JM, Ruiz C, Melguizo-Rodriguez L. SARS-CoV-2 infection: The role of cytokines in COVID-19 disease. Cytokine Growth Factor Rev. 2020;54:62–75. Epub 2020/06/10. doi: 10.1016/j.cytogfr.2020.06.001 ; PubMed Central PMCID: PMC7265853. 32513566 PMC7265853

[26] Duncan DJ, Yang Z, Hopkins PM, Steele DS, Harrison SM. TNF-alpha and IL-1beta increase Ca2+ leak from the sarcoplasmic reticulum and susceptibility to arrhythmia in rat ventricular myocytes. Cell Calcium. 2010;47(4):378–86. Epub 2010/03/17. doi: 10.1016/j.ceca.2010.02.002 ; PubMed Central PMCID: PMC2877880. 20227109 PMC2877880

[27] Del Valle DM, Kim-Schulze S, Huang HH, Beckmann ND, Nirenberg S, Wang B, et al. An inflammatory cytokine signature predicts COVID-19 severity and survival. Nat Med. 2020;26(10):1636–43. Epub 2020/08/26. doi: 10.1038/s41591-020-1051-9 ; PubMed Central PMCID: PMC7869028. 32839624 PMC7869028

[28] Liu SJ, Zhou W, Kennedy RH. Suppression of beta-adrenergic responsiveness of L-type Ca2+ current by IL-1beta in rat ventricular myocytes. Am J Physiol. 1999;276(1):H141–8. Epub 1999/01/14. doi: 10.1152/ajpheart.1999.276.1.H141 . 9887027

[29] Mitrokhin VM, Mladenov MI, Kamkin AG. IL-1 provokes electrical abnormalities in rat atrial myocardium. Int Immunopharmacol. 2015;28(1):780–4. Epub 2015/08/19. doi: 10.1016/j.intimp.2015.08.006 . 26283592

[30] Garcia-Zamora S, Lee S, Haseeb S, Bazoukis G, Tse G, Alvarez-Garcia J, et al. Arrhythmias and Electrocardiographic findings in Coronavirus disease 2019: a systematic review and meta-analysis. Pacing Clin Electrophysiol. 2021. Epub 2021/04/24. doi: 10.1111/pace.14247. 33890684 PMC8250376

[31] Huke S, Knollmann BC. Increased myofilament Ca2+-sensitivity and arrhythmia susceptibility. J Mol Cell Cardiol. 2010;48(5):824–33. Epub 2010/01/26. doi: 10.1016/j.yjmcc.2010.01.011 ; PubMed Central PMCID: PMC2854218. 20097204 PMC2854218

[32] Zhu X, Bernecker OY, Manohar NS, Hajjar RJ, Hellman J, Ichinose F, et al. Increased leakage of sarcoplasmic reticulum Ca2+ contributes to abnormal myocyte Ca2+ handling and shortening in sepsis. Crit Care Med. 2005;33(3):598–604. Epub 2005/03/09. doi: 10.1097/01.ccm.0000152223.27176.a6 . 15753753

[33] Katia F, Myriam DP, Ucciferri C, Auricchio A, Di Nicola M, Marchioni M, et al. Efficacy of canakinumab in mild or severe COVID-19 pneumonia. Immun Inflamm Dis. 2021;9(2):399–405. Epub 2021/01/20. doi: 10.1002/iid3.400 ; PubMed Central PMCID: PMC8013503. 33465283 PMC8013503

[34] Sheng CC, Sahoo D, Dugar S, Prada RA, Wang TKM, Abou Hassan OK, et al. Canakinumab to reduce deterioration of cardiac and respiratory function in SARS-CoV-2 associated myocardial injury with heightened inflammation (canakinumab in Covid-19 cardiac injury: The three C study). Clin Cardiol. 2020;43(10):1055–63. Epub 2020/08/25. doi: 10.1002/clc.23451 ; PubMed Central PMCID: PMC7461303. 32830894 PMC7461303

